# Evaluation of food insecurity and associated factors in women of childbearing age: A community‐based study from Turkey

**DOI:** 10.1002/fsn3.3743

**Published:** 2023-10-10

**Authors:** Gizem Aytekin Sahin, Ozge Mengi Celik

**Affiliations:** ^1^ Department of Nutrition and Dietetics, Faculty of Health Sciences Nuh Naci Yazgan University Kayseri Turkey; ^2^ Department of Nutrition and Dietetics, Gulhane Faculty of Health Sciences University of Health Sciences Ankara Turkey

**Keywords:** childbearing age women, food insecurity, Mediterranean diet

## Abstract

Food security is an important factor in ensuring a healthy diet. However, it has been reported that women are more vulnerable to food insecurity than men in many countries. In addition, there is little evidence that Mediterranean diet (MD) adherence is associated with a lower risk of food insecurity. Therefore, this study aimed to evaluate food insecurity and associated factors in women of childbearing age. In addition, women's adherence to MD and its relationship with food insecurity were evaluated. This descriptive and cross‐sectional study was conducted with 2675 adult women aged 18–49. The demographic characteristics, anthropometric measurements, nutritional habits, compliance with the MD, and the level of food insecurity were evaluated using the structured, self‐administered, web‐based questionnaire form. The mean age of the individuals was 29.5 ± 10.35 years. 21.6% of individuals had food insecurity, and 5.2% had severe food insecurity. 36.4% of the individuals adhere to the MD. There was a statistically significant difference between the individuals with and without food security regarding age, marital status, and income status (*p* < .05). However, there was no statistically significant difference in terms of the Mediterranean diet adherence score (MEDAS) and MEDAS classification between individuals with and without food security (*p* > .05). It was determined that age, marital status, and income status affected food insecurity (*R* ^2^ = 0.374; *p* < .001). Also, it was determined that age, number of main meals and snacks, and income status affected adherence to MD (*R* ^2^ = 0.286; *p* < .001). In conclusion, considering the fragility of women in food insecurity, more comprehensive studies should be conducted in this area to better define the factors associated with food insecurity in women and inform policymakers. In addition, the results of this study can shed light on comprehensive intervention studies in this area.

## Graphical abstract

This descriptive and cross‐sectional study was conducted with 2675 adult women aged 18–49. There was a statistically significant difference between the individuals with and without food security regarding age, marital status, and income status. There was no statistically significant difference in terms of the Mediterranean diet adherence score (MEDAS) and MEDAS classification between individuals with and without food security (*p* > .05). It was determined that age, marital status, and income status had an effect on food insecurity.

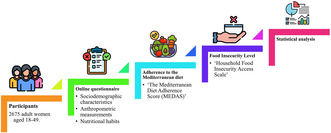

## 1 INTRODUCTION

Food insecurity refers to the lack of access to sufficient, affordable, and nutritious food or the inability to obtain food in a socially acceptable manner. This condition is characterized by inadequate food consumption, limited food accessibility, and vulnerability to subsistence strategies that cannot withstand unexpected events (Samim et al., 2021).

Although there have been some efforts to address global malnutrition and hunger, food insecurity is still a major concern worldwide (Samim et al., 2021). According to the United Nations Food and Agricultural Organization (FAO) 2022 reports, the worldwide prevalence of moderate food insecurity remained stable after a significant increase in 2020. However, severe food insecurity continued to rise, and approximately 2.3 billion individuals worldwide encountered moderate or severe food insecurity in 2021, and 11.7% of the global population faced severe levels of food insecurity (Burki, 2022).

Since Turkey is located in the Middle East, where various civil wars and political instability are experienced, it is crucial to evaluate food security regularly (Keskin & Demirbas, 2022). In this context, the data from the Global Hunger Index (GHI) show that Turkey has improved its situation in terms of food security. According to these data, while the hunger rate in Turkey was 10.1% in 2000, it has been reported to be below 5% since 2007 (Concern Worldwide & Welthungerhilfe, 2022). However, according to the Global Food Security Index (GFSI), Turkey ranks 49th out of 113 countries (Economist Impact, 2022). In previous studies with relatively small sample sizes in Turkey, the food insecurity rate was found to be 21.9%–29.9% in older people (Simsek et al., 2013; Tari Selcuk et al., 2023), 21.8% in households with children (Bulucu Büyüksoy et al., 2021), and 33% in university students (Ni̇yaz, 2020). Considering these data, it can be said that food insecurity continues to be an important problem in Turkey and needs further research. However, there is no data on the rate of food insecurity among women of childbearing age in Turkey.

It is widely acknowledged that women play a crucial role in managing food in the household. In addition, many studies have reported that women are more vulnerable to food insecurity than men in both developed and developing countries (Grimaccia & Naccarato, 2022). Furthermore, food insecurity is a significant public health concern that often leads to unhealthy eating habits and chronic diseases. Various studies have demonstrated that food insecurity is linked to unhealthy dietary patterns, such as reduced consumption of fruits, vegetables, and dairy products and increased intake of energy‐dense foods (Gregório et al., 2018; Hanson et al., 2007). In addition, it has been reported that food‐insecure individuals consume inadequate amounts of various nutrients (Gundersen & Seligman, 2017). Moreover, food insecurity leads to potential health consequences, hunger, and malnutrition, such as obesity, cancer, diabetes, and psychosocial stress (Lee & Frongillo, 2001). Therefore, investigating food insecurity for women of childbearing age is also essential for the health of future generations.

The Mediterranean diet (MD) is a traditional dietary model characterized by a high intake of fruits and vegetables, whole grains, legumes, nuts, olive oil, and a moderate meat intake. According to several studies, following MD can decrease the risk of nutrition‐related chronic diseases such as obesity, cardiovascular diseases, diabetes, and cancer (Guasch‐Ferré et al., 2017). In addition, the MD is described by the Food and Agriculture Organization (FAO) as a sustainable diet model, and several studies have reported that adherence to the MD could significantly decrease the environmental impact of the diet (Fresán et al., 2018; Tilman & Clark, 2014). Various tools, such as frequency questionnaires, food diaries, food and dietary assessment surveys, or scales, are used to evaluate adherence to the MD (Hidalgo‐Mora et al., 2020). The Mediterranean diet adherence score (MEDAS), developed with contributions from the PREDIMED study, is one of the most well‐accepted and widely used tools (Schröder et al., 2011).

Various studies have found a relationship between different diet quality scores (such as Total Diet Score, Healthy Eating Index, Index‐Member Dietary Diversity Score, Alternative Healthy Eating Index, Recommended Food Score, Minimum Dietary Diversity Score for Women) and food insecurity (Becquey et al., 2010; Kehoe et al., 2021; Muhammad et al., 2019; Russell et al., 2016). The Mediterranean diet score is one of them, and studies have also shown a negative relationship between adherence to the MD and food insecurity in different populations (Gregório et al., 2018; Naja et al., 2020; Theodoridis et al., 2018). In addition, as far as we know, there is no study on the relationship between adherence to the MD and food insecurity in women of childbearing age.

Furthermore, although gender is an important determinant of food insecurity, it is essential to investigate the factors that cause it specifically for each population, considering the variability of geographic distribution and the complex relationships with economic and social factors (Grimaccia & Naccarato, 2022). However, the issue of food insecurity among childbearing‐age women has yet to receive adequate attention, and factors associated with food insecurity among childbearing‐age women could not be fully understood. Therefore, this study aimed to evaluate food insecurity and associated factors in women of childbearing age. In addition, women's adherence to MD and its relationship with food insecurity were evaluated.

## 2 MATERIALS AND METHODS

This descriptive and cross‐sectional study was conducted with 2675 adult women aged 18–49 between December 2022 and April 2023. Research data were collected with the help of a web‐based questionnaire. The study sample was formed by the individuals who ticked the ‘I consent to participate in this study voluntarily’ tab at the beginning of the form and filled out the questionnaire completely. Before starting the study, ethical approval was obtained with decision number 220/17 dated 15.11.2021 from the Trakya University Faculty of Medicine Dean's Office of Ethics Committee for Non‐Invasive Scientific Research. All procedures in the study were carried out in accordance with the Declaration of Helsinki. The demographic characteristics (age, education level, working status, marital status, and income status), anthropometric measurements (body weight and height), nutritional habits (number of main meals and snacks), compliance with the MD, and the level of food insecurity were questioned through the questionnaire form.

### 2.1 Anthropometric measurements

Anthropometric measurements (body weight and height) were taken based on individuals' self‐reports. Individuals were informed about how to take anthropometric measurements in the questionnaire form. The body mass index (BMI) value was calculated by dividing the body weight by the square of the height. BMI below 18.50 kg/m^2^ was classified as underweight, between 18.50–24.99 kg/m^2^ as normal, 25.0–29.99 kg/m^2^ as overweight, and above 30.0 kg/m^2^ as obese (Madden & Smith, 2016).

### 2.2 Food insecurity

Individuals’ food insecurity level was evaluated with the ‘Household Food Insecurity Access Scale’ (Coates et al., 2007). The Turkish validity and reliability study of the scale was conducted in 2018 (Bor, 2018). There are 9 questions on the scale that evaluate the situation of food insecurity in the last month. The maximum score that can be obtained from the scale is 27, and the minimum score is 0. The increase in the total score indicates that the food insecurity felt by the household is severe. A low score indicates that the household's food insecurity level is also low. Individuals’ food insecurity status was classified into four groups according to the scores they got from the questions: food insecurity, mild food insecurity, moderate food insecurity, and severe food insecurity (Coates et al., 2007).

### 2.3 The Mediterranean diet adherence score

The MEDAS was used to determine the participants' features related to the MD pattern. The scale consists of 14 questions about food intake habits and food consumption frequency that are typical and non‐typical of the MD. Responses aligned with adherence to the MD are assigned 1 point, whereas those that did not are given 0 points. The total score ranges between 1 and 14, and a total score of 7 or higher shows that the subject adheres to the MD appropriately (García‐Conesa et al., 2020; Guasch‐Ferré et al., 2017).

### 2.4 Statistical analysis

The Statistical Package for the Social Sciences (SPSS, version 22.0) software was used for all analyses. Data were evaluated with descriptive statistics such as mean, standard deviation, number, and percentage. Distribution analysis of the data was performed using the histogram, coefficient of variation ratio, skewness, kurtosis, and Kolmogorov–Smirnov tests. Differences in mean values between groups (food insecurity and compliance with the MD) were evaluated with the independent t test. Chi‐square analysis was used to compare qualitative data between groups (food insecurity and compliance with the MD). Regression analysis was performed for the prediction of food insecurity and compliance with the MD. A *p*‐value of <.05 was considered to be statistically significant.

## 3 RESULTS

The general characteristics of the individuals are given in Table 1. The mean age of the individuals was 29.5 ± 10.35 years, and the mean BMI was 23.7 ± 4.65 kg/m^2^. Most individuals (68.6%) were university graduates. While 60.0% of individuals were of normal weight according to their BMI classification, 21.3% were overweight, and 10.5% were obese. The income of 20.4% of individuals was less than their expenses. 39.7% of the individuals were married and 33.7% were working in a job. The mean number of main meals was 2.4 ± 0.52, and the mean number of snacks was 1.2 ± 1.11. While 21.6% of individuals had food insecurity, 5.2% had severe food insecurity, and 36.4% adhered to the MD.

**TABLE 1:** General characteristics of individuals.

Variables	$\bar{X}\pm \mathrm{SD}$
Age (years)	29.5 ± 10.35
BMI (kg/m^2^)	23.7 ± 4.65
Number of main meals	2.4 ± 0.52
Number of snacks	1.2 ± 1.11
Household Food Insecurity Access Scale Score	0.9 ± 2.74
MEDAS	5.8 ± 2.23

	Number (%)
Education level	
Primary school	145 (5.4)
Middle school	92 (3.4)
High school	461 (17.2)
University	1835 (68.6)
Master's degree/doctorate	142 (5.3)
Marital status	
Married	1062 (39.7)
Unmarried	1613 (60.3)
Working status	
Yes	901 (33.7)
No	1774 (66.3)
Income status	
Income more than expenses	758 (28.3)
Income equal to expenses	1370 (51.2)
Income less than expenses	547 (20.4)
BMI classification	
Underweight (<18.50 kg/m^2^)	219 (8.2)
Normal (18.50–24.99 kg/m^2^)	1605 (60.0)
Overweight (25.00–29.99 kg/m^2^)	571 (21.3)
Obese (≥30.0 kg/m^2^)	280 (10.5)
Evaluation of the food insecurity	
Food security	2098 (78.4)
Food insecurity	577 (21.6)
Mildly food insecure	239 (8.9)
Moderately food insecure	200 (7.5)
Severely food insecure	138 (5.2)
MEDAS classification	
Compliance with the MD (≥7 points)	974 (36.4)
No compliance with the MD (<7 points)	1701 (63.6)

Abbreviations: BMI, body mass index; MD, Mediterranean diet; MEDAS, The Mediterranean diet adherence score.

The evaluation of food insecurity according to the demographic characteristics of individuals is given in Table 2. There was a statistically significant difference between the individuals with and without food security regarding age, marital status, and income status (*p* < .05). There was no statistically significant difference between individuals with and without food security in terms of MEDAS and MEDAS classification, number of main meals and snacks, education level, working status, BMI, and BMI classification (*p* > .05).

**TABLE 2:** Evaluation of food insecurity according to the demographic characteristics of individuals.

Variables	Food insecurity status		*p*
	Food security	Food insecurity	
Age (years)	29.8 ± 10.46	28.6 ± 9.91	*p* = .014^a^, *
Number of main meals	2.4 ± 0.52	2.3 ± 0.55	*p* = .083^a^
Number of snacks	1.1 ± 1.00	1.1 ± 1.20	*p* = .791^a^
Education level			*p* = .633^b^
Primary school	120 (5.7)	25 (4.3)	
Middle school	75 (3.6)	17 (2.9)	
High school	358 (17.1)	103 (17.9)	
University	1432 (68.3)	403 (69.8)	
Master's degree/doctorate	113 (5.4)	29 (5.0)	
Marital status			*p* = .007^b^ ^,^ *
Married	859 (40.9)	203 (35.2)	
Unmarried	1239 (59.1)	374 (64.8)	
Working status			*p* = .208^b^
Yes	698 (33.3)	203 (35.2)	
No	1400 (66.7)	374 (64.8)	
Income status			*p* < .001^b^ ^,^ *
Income more than expenses	666 (31.7)	92 (15.9)	
Income equal to expenses	1072 (51.1)	298 (51.6)	
Income less than expenses	360 (17.2)	187 (32.4)	
BMI (kg/m^2^)	23.7 ± 4.61	23.8 ± 4.79	*p* = .596^a^
BMI classification (number, %)			*p* = .939^b^
Underweight (<18.5 kg/m^2^)	171 (8.2)	48 (8.3)	
Normal (18.5–24.99 kg/m^2^)	1265 (60.3)	340 (58.9)	
Overweight (≥25.00–29.99 kg/m^2^)	443 (21.1)	128 (22.2)	
Obese (≥30.0 kg/m^2^)	219 (10.4)	61 (10.6)	
MEDAS	5.9 ± 2.27	5.8 ± 2.24	*p* = .791^a^
MEDAS classification			*p* = .129^b^
Adherence to the MD	776 (37.0)	198 (34.3)	
No adherence to the MD	1322 (63.0)	379 (65.7)	

Abbreviations: BMI, body mass index; MD, Mediterranean diet; MEDAS, The Mediterranean diet adherence score.

^a^ Independent *t* test.

^b^ Chi‐square test.

* *p* < .05.

When the factors that could affect the Household Food Insecurity Access Scale Score were evaluated with linear regression analysis, the model was deemed important (*R* ^2^ = 0.374; *p* < .001). Age, marital status, and income were associated with food insecurity (*p* < .05) (Table 3).

**TABLE 3:** Linear regression model for food insecurity prediction.

Model	Household Food Insecurity Access Scale Score		
	Beta	*T*	*p*‐value
Age (years)	−0.258	−10.391	<.001*
Marital status	−0.204	−9.327	<.001*
Income status	−0.154	−8.027	<.001*
		*R* ^2^ = 0.374; *p* < .001*	

*Note*: Variable values: Marital status (married = 1, unmarried = 0), income status (income less than expenses = 1, income equal to expenses = 2, income more than expenses = 3).

* *p* < .05.

The evaluation of adherence to the MD according to the demographic characteristics of individuals is given in Table 4. A statistically significant difference was found between individuals who did and did not adhere to the MD in age, number of main meals and snacks, and income status (*p* < .05). There was no statistically significant difference between these two groups in terms of Household Food Insecurity Access Scale Score, education level, marital status, working status, BMI, and BMI classification (*p* > .05).

**TABLE 4:** Evaluation of adherence to the Mediterranean diet according to the demographic characteristics of individuals.

Variables	Adherence to the MD	No adherence to the MD	*p*
Age (years)	30.1 ± 10.68	29.2 ± 10.15	*p* = .026^a^ ^,^ *
Number of main meals	2.5 ± 0.53	2.3 ± 0.52	*p* < .001^a^ ^,^ *
Number of snacks	1.2 ± 1.17	1.1 ± 1.07	*p* = .032^a^ ^,^ *
Education level			*p* = .368^b^
Primary school	60 (6.2)	85 (5.0)	
Middle school	36 (3.7)	56 (3.3)	
High school	180 (18.5)	281 (16.5)	
University	647 (66.4)	1188 (69.8)	
Master's degree/doctorate	51 (5.2)	91 (5.3)	
Marital status			*p* = .085^b^
Married	408 (41.9)	654 (38.4)	
Unmarried	566 (58.1)	1047 (61.6)	
Working status			*p* = .236^b^
Yes	337 (34.6)	564 (33.2)	
No	637 (65.4)	1137 (66.8)	
Income status			*p* = .010^b^ ^,^ *
Income more than expenses	308 (31.6)	450 (26.5)	
Income equal to expenses	486 (49.9)	884 (52.0)	
Income less than expenses	180 (18.5)	367 (21.6)	
BMI (kg/m^2^)	23.8 ± 4.91	23.7 ± 4.50	*p* = .282^a^
BMI classification (number, %)			*p* = .752^b^
Underweight (<18.5 kg/m^2^)	77 (7.9)	142 (8.3)	
Normal (18.5–24.99 kg/m^2^)	580 (59.5)	1025 (60.3)	
Overweight (≥25.00–29.99 kg/m^2^)	207 (21.3)	364 (21.4)	
Obese (≥30.0 kg/m^2^)	110 (11.3)	170 (10.0)	
Household Food Insecurity Access Scale Score	0.9 ± 2.73	1.0 ± 2.74	*p* = .884^a^

Abbreviations: BMI, body mass index; MD, Mediterranean diet.

^a^ Independent *t* test.

^b^ Chi‐square test.

* *p* < .05.

When the factors that could affect the MEDAS were evaluated with linear regression analysis, the model was deemed important (*R* ^2^ = 0.286; *p* < .001). It was determined that age, number of main meals and snacks, and income status affected adherence to the MD (*p* < .05) (Table 5).

**TABLE 5:** Linear regression model for compliance with the Mediterranean diet prediction.

Model	MEDAS		
	Beta	*t*	*p*‐value
Age (years)	0.190	8.037	<.001*
Number of main meals	0.196	8.087	<.001*
Number of snacks	0.112	6.487	<.001*
Income status	0.260	10.774	<.001*
		*R* ^2^ = 0.286; *p* < .001*	

*Note*: Variable values: Income status (income less than expenses = 1, income equal to expenses = 2, income more than expenses = 3).

* *p* < .05.

## 4 DISCUSSION

The present study determined that age, marital status, and income status were effective factors in food insecurity. However, unlike some studies, there was no statistically significant difference between women with and without food security regarding adherence to the MD. These results are important as they shed light on the factors affecting food insecurity in women of childbearing age with a large sample.

Women within the childbearing age range may face a higher risk of experiencing negative health consequences due to the added physical requirements of pregnancy and lactation (Rai et al., 2015). Therefore, adequate and balanced nutrition before and during pregnancy is also important in obtaining a healthy pregnancy and birth result (Zhang et al., 2008). Food security is an important condition for ensuring a healthy diet. However, it has been reported that women are more vulnerable to food insecurity than men in both developed and developing countries (Grimaccia & Naccarato, 2022). However, as far as we know, there is no study investigating food insecurity in women of childbearing age in Turkey.

Studies have found that food insecurity in the general population in Turkey is between 22% and 69% (Eştürk, 2015; Simsek et al., 2013; Tari Selcuk et al., 2023). In this study, the food insecurity rate was found to be 21.6% among childbearing‐age women. The food insecurity rate in women in similar age groups in different countries is between 8% and 46% (Basiotis & Lino, 2003; Hilmers et al., 2014; Zizza et al., 2008). The differences in the methods and the study sample may have caused the results to differ.

Although age has been reported as one of the factors associated with the risk of food insecurity in the literature (Alvares & Amaral, 2014; Miller et al., 2020; Tari Selcuk et al., 2023), there is no consistency between the results. The inconsistencies in the results may be due to the differences in age groups in the studies. In our study, the risk of food insecurity decreased as age increased. Young women have yet to achieve economic independence or, if unmarried, do not have sufficient socio‐economic power, which may have caused this situation due to their limited access to food.

In our study, marital status is another factor related to food insecurity in women of childbearing age. It has been determined that married women's food insecurity risk is lower than that of single women. Similarly, in the literature, married individuals’ food insecurity risk was lower than that of unmarried individuals (Grimaccia & Naccarato, 2022; Hanson et al., 2007). Marriage provides increased family income and wealth. Spouses also experience economic hardships together, and sharing resources may reduce food insecurity risk (Hanson et al., 2007).

Food insecurity means the lack of access to adequate, affordable, and nutritious food or the inability to obtain food in a socially acceptable way (Samim et al., 2021). Therefore, income level has been reported as an important factor associated with food insecurity in the literature (Committee on World Food Security, 2020; Gregório et al., 2018; Grimaccia & Naccarato, 2022). Moreover, studies, unfortunately, emphasize that women have a higher risk of food insecurity in terms of low‐income status (Grimaccia & Naccarato, 2022; Ivers & Cullen, 2011). As expected in this study, having a lower income status was associated with an increased risk of food insecurity.

The MD is one of the healthiest eating patterns, with low saturated fat, a high content of vegetables, fruits, and dietary fiber, and a wide range of health benefits (Guasch‐Ferré & Willett, 2021). It is also considered as sustainable diet model (Berry, 2019). The present study provides information about the factors that affect the adherence of women of childbearing age to the MD. Age, number of main meals, number of snacks, and income status were found to be factors affecting adherence to the MD.

According to the literature, age has been found to be a significant factor in determining healthy behaviors (Caparello et al., 2020). Previous studies have suggested that younger individuals have lower adherence to the MD (Grosso & Galvano, 2016; Iaccarino Idelson et al., 2017). Similarly, our study observed that as the women's age increased, the scores of adherence to the MD increased. Aging can bring many health problems, which may lead people to eat healthier. The MD includes nutrients such as antioxidants, healthy fats, whole grains, fruits, and vegetables. Consumption of these nutrients could prevent the development of some age‐related health problems. Therefore, women may have adapted to the MD to improve or maintain their health as they age.

The present study found a positive relationship between the number of main meals and snacks and the scores of adherence to the MD. The MEDAS, which we use to evaluate adherence to MD, is a scale that includes daily consumption amounts of foods such as vegetables, fruits, and legumes (García‐Conesa et al., 2020). As the number of main meals and snacks increased, women may have tended to consume a broader range of foods. In addition, because the MD includes a variety of food groups, these women may have had more opportunities to diversify their diets and try different foods, which may have increased their MEDAS.

Income status is a factor that impacts participants' dietary habits. In addition, several studies reported that people with higher incomes showed better adherence to the MD, and having a higher income was positively associated with a higher score of adherence to the MD (Biasini et al., 2021; Cavaliere et al., 2018). The results of our study are consistent with these findings.

Generally, adherence to the MD has been associated with a lower risk of food insecurity (Naja et al., 2020; Theodoridis et al., 2018). In this study, unlike other studies, no relationship was found between participants’ adherence to MD and food insecurity. In addition, the participants in the food security group and the food insecurity group had similar MEDAS and rates of adherence to the MD. These results can be interpreted in two ways. First, the ‘Household Food Insecurity Access Scale’ could not precisely measure access to healthy and nutritious food. The scale generally includes questions about access to adequate food, which may not always mean access to healthy food. Second, in this study, there is no information about the awareness or knowledge level of the participants about healthy eating. Therefore, participants in the food security group may have preferred unhealthy foods and an unhealthier diet. However, cross‐sectional studies are not sufficient to explain more clearly whether there is a relationship between adherence to the MD and food insecurity, and empirical studies are needed on this subject.

The present study has some strengths. First, the study was conducted using a large sample size. Second, studies investigating food insecurity in women of childbearing age are relatively limited in the literature, and this study is the first in Turkey, to our knowledge. However, this study has several limitations. First, the lack of a causal link due to its cross‐sectional nature is a limitation of the study. Second, we evaluated food insecurity and adherence to MD based on individual self‐perception. In addition, the ‘Household Food Insecurity Access Scale’ cannot provide an understanding of how food insecurity affects women of childbearing age differently, regardless of the household. Nevertheless, the findings of our study will shed light on future studies.

## 5 CONCLUSION

In conclusion, the present study determined that more than one‐fifth of women of childbearing age are faced with food insecurity. In addition, age, marital status, and income status were found to be factors associated with food insecurity. While age, number of main meals and snacks, and income status affect adherence to MD, contrary to the literature, there was no relationship between adherence to MD and food insecurity.

At this point, the food aid from the governments after the sensitive groups are identified and evaluated in terms of food security may be beneficial in reducing the rates of food insecurity. In addition, educating women on food purchasing, food preparation, and cooking by experts may reduce food insecurity rates and increase adherence to the MD. It is essential to solve the problem of food insecurity among women of childbearing age for the continuation of healthy generations. In addition, empirical studies are needed to reveal the relationship more clearly between adherence to the MD and food insecurity. Considering the fragility of women in food insecurity, it is crucial to carry out comprehensive studies to better define the factors associated with food insecurity in women and to inform policymakers on this issue.

## AUTHOR CONTRIBUTIONS

**Gizem Aytekin Sahin:** Conceptualization (equal); data curation (equal); formal analysis (equal); investigation (equal); methodology (equal); project administration (equal); visualization (equal); writing – original draft (equal); writing – review and editing (equal). **Ozge Mengi Celik:** Conceptualization (equal); data curation (equal); formal analysis (equal); investigation (equal); methodology (equal); project administration (equal); writing – original draft (equal); writing – review and editing (equal).

## CONFLICT OF INTEREST STATEMENT

The authors declare that there are no conflicts of interest.

## Data Availability

The data that support the findings of this study are available from the corresponding author upon reasonable request.
